# A machine learning analysis of perceived healthcare access barriers among women of reproductive age in Sub-Saharan Africa: using demographic and health surveys

**DOI:** 10.1016/j.xagr.2026.100668

**Published:** 2026-07-03

**Authors:** Fentahun Bikale Kebede, Abraham Keffale Mengistu

**Affiliations:** 1 Strategic Affairs Executive Office, Ministry of Health, Addis Ababa, Ethiopia (Kebede); 2 Department of Health Informatics, College of Medicine and Health Sciences, Debre Markos University, Debre Markos, Ethiopia (Mengistu)

**Keywords:** machine learning, perceived healthcare access, SHAP, Sub-Saharan Africa, women’s health

## Abstract

**BACKGROUND:**

In Sub-Saharan Africa (SSA), women of reproductive age face significant barriers to accessing healthcare, undermining progress towards Universal Health Coverage. Understanding the complex determinants of this multifaceted problem requires advanced analytical approaches.

**OBJECTIVE:**

This study aimed to identify and rank the key predictors of perceived healthcare access barriers among women in SSA using a multi-country dataset and an interpretable machine learning (ML) framework.

**Study Design:**

We analyzed recent Demographic and Health Survey data from 28 SSA countries (n=467,965 women). A composite measure of healthcare access barriers was used as the outcome. After preprocessing and feature selection, eight ML models were trained and evaluated. The best-performing model was interpreted using SHapley Additive exPlanations (SHAP).

**RESULTS:**

The pooled prevalence of perceived healthcare access barriers ranges from 17.8% (95% CI, 17.0–18.6%) in South Africa to 72.1% (95% CI, 71.6–72.7%) in Malawi. The Light Gradient Boosting Machine (LightGBM) was the top-performing model (Accuracy:65.2%, F1-score: 72.8%, ROC-AUC:68.2%, and PR-AUC: 72.9%). SHAP analysis identified the wealth index as the most powerful predictor, followed by use of internet, media exposure, mobile phone ownership, and educational level. Lower socioeconomic status and limited access to information and communication technologies were strongly associated with greater access barriers

**CONCLUSION:**

The findings underscore that socioeconomic and digital inclusion factors are paramount in determining healthcare access. This highlights the need for integrated strategies that go beyond health-sector solutions to address underlying social and economic determinants. This study provides an evidence-based, actionable ranking of the determinants of perceived healthcare access problems. The results can inform targeted policies on poverty alleviation, digital inclusion, and education to reduce disparities and advance equitable healthcare access for women in SSA.

## Highlights

• The prevalence of perceived barriers to healthcare access ranges from 17.8% (95% CI, 17.0%–18.6%) in South Africa to 72.1% (95% CI, 71.6%–72.7%) in Malawi.
• LightGBM showed the best predictive performance (Accuracy: 65.2%, F1-score: 72.8%, ROC-AUC: 68.2%, and PR-AUC: 72.9%).
• Wealth index was the strongest predictor, followed by internet use, media exposure, mobile phone ownership, and education.
• The study provides a machine-learning-based, interpretable ranking of determinants of healthcare access barriers,
• The study highlights socioeconomic status and digital inclusion as critical factors in informing policies beyond the health sector.

**AJOG Global Reports at a Glance**

**Why was this study conducted?**

To identify and prioritize the determinants of perceived barriers to healthcare access among women in sub-Saharan Africa using interpretable machine learning models.

**Key findings**

The prevalence of perceived barriers to healthcare access ranges from 17.8% (95% CI, 17.0-18.6%) in South Africa to 72.1% (95% CI, 71.6-72.7%) in Malawi.

LightGBM showed the best predictive performance (Accuracy:65.2%, F1-score: 72.8%, ROC-AUC:68.2%, and PR-AUC: 72.9%).

Wealth index was the strongest predictor, followed by internet use, media exposure, mobile phone ownership, and education.

**What does this study add to what is known?**

Provides a machine-learning-based, interpretable ranking of determinants of healthcare access barriers.

Highlights socioeconomic status and digital inclusion as critical factors in informing policies beyond the health sector.

## Introduction

Universal access to acceptable, affordable, and quality healthcare remains one of the fundamental pillars of the Sustainable Development Goals (SDGs), in particular SDG 3, and is central to the global pursuit of Universal Health Coverage. Despite this, progress has been uneven and slowed over the last decade. According to a report by the World Health Organization (WHO) and the World Bank in 2021, it is estimated that 4.5 billion people worldwide lack full coverage of essential health services, with nearly 2 billion facing financial hardship due to out-of-pocket health expenditures.^1^, ^2^, ^3^ This burden comes disproportionately from sub-Saharan Africa, where more than eleven million people have been pushed below the poverty line each year due to healthcare costs, and the ability to access basic services remains exceptionally low.^4^, ^5^, ^6^, ^7^, ^8^ Healthcare access is a multifaceted concept, including affordability, acceptability, availability, and use of services for optimal health outcomes.^9^, ^10^, ^11^, ^12^, ^13^, ^14^

The crisis of health care access is particularly acute for women of reproductive age in the challenging context of SSA.^15^, ^16^, ^17^, ^18^ Educational and income differentials, accompanied by limitations in social power, are deeply entrenched and severely restrict their seeking and receiving care.^15^, ^19^, ^20^, ^21^, ^22^ Deep-seated cultural and gender norms further exacerbate these barriers, where decision-making autonomy, mobility, and control over financial resources are severely restricted.^23^, ^24^, ^25^, ^26^ This has many consequences, as women bear a disproportionate share of reproductive and caregiving responsibilities and face especially significant obstacles, such as financial constraints, long distances to facilities, lack of transportation, and the social impediment of not wanting to go to a clinic alone. These embedded, complex issues reflect a critical public health challenge that threatens maternal health outcomes, obstructs progress toward the SDGs, and perpetuates cycles of gender inequality across the continent.^27^, ^28^, ^29^, ^30^, ^31^

Determinants of access to health care have been the subject of a great deal of research, and many social, demographic, and economic factors have been identified. Key variables consistently found to be associated with barriers to access include: geographic region, residence in rural areas, low maternal and paternal education levels, poverty (low wealth index), lack of health insurance, and limited exposure to mass media.^16^^,^^17^^,^^32^^,^^33^ These issues have been explored empirically at both the national and regional levels within SSA.^18^ While such studies are truly enlightening, they tend to depend on the traditional statistical tools commonly used, such as regression analysis, which is superb for specific hypothesis testing and causal relationships, but perhaps at a disadvantage when handling complex, high-dimensional interactions in big, multicountry datasets. With healthcare access problems for women in SSA being some of the most persistent and complex, there is a growing imperative for a comprehensive, multicountry analysis that can both capture broad contextual variations and identify the most influential predictors. This study addresses this gap by leveraging the predictive power of ML on recent, pooled DHS data from 28 SSA countries. While traditional regression methods remain vital for causal inference, ML algorithms have many unique advantages for this task, including robust handling of complex, nonlinear relationships between variables; inherent feature selection capabilities; and high predictive accuracy. The application of these advanced techniques in the current study enables a move beyond the mere identification of associated factors to the ranking of their relative importance and the building of a predictive model, offering an understanding of the problem that is both more nuanced and actionable. This study will provide an updated, evidence-based roadmap for policymakers and health planners. The three major objectives of the study are: 1) to estimate the pooled and country-specific prevalence of perceived healthcare access barriers among women of reproductive age in SSA; 2) to identify and rank the key determinants of the access problem using advanced ML models; and 3)to develop and validate a predictive model to help stakeholders proactively identify the high-risk populations in advance and prioritize targeted interventions when and where they are most needed. The findings are expected to inform the design of more effective, data-driven strategies to overcome access barriers and accelerate progress toward universal health coverage and gender equity in sub-Saharan Africa.

## Methods

### Data sources

This study uses the most recent Demographic and Health Survey (DHS) data from 28 countries in SSA from 2015 to 2024, including Cameroon, DR Congo, and Gabon from Central Africa; Ethiopia, Kenya, Madagascar, Malawi, Mozambique, Rwanda, Tanzania, and Uganda from East Africa; Angola, Burundi, Lesotho, South Africa, and Zambia from Southern Africa; and Benin, Burkina Faso, Côte d'Ivoire, Ghana, Guinea, Liberia, Mali, Mauritania, Nigeria, Senegal, Sierra Leone, Gambia, from West Africa (Table 1).

**Table 1:** Pooled demographic and health survey (DHS) data from 28 Sub-Saharan African countries.

Country	Year of survey	Total weighted sample size
**Central Africa**		**53303**
Cameroon	2018/19	14677
DR, Congo	2023/24	27583
Gabon	2019/21	11043
**East Africa**		**152847**
Ethiopia	2016	15683
Kenya	2022	32156
Madagascar	2021	18869
Malawi	2015/16	24562
Mozambique	2022/23	13183
Rwanda	2019/20	14634
Tanzania	2022	15254
Uganda	2016	18506
**Southern Africa**		**60258**
Angola	2015/16	14379
Burundi	2016/17	17269
Lesotho	2023/24	6413
South Africa	2016	8514
Zambia	2018/19	13683
**West Africa**		**201557**
Benin	2017/18	15928
Burkina Faso	2021	17659
Côte d'Ivoire	2021	14877
Ghana	2022/23	15014
Guinea	2018	10874
Liberia	2019/20	8065
Mali	2023/24	20354
Mauritania	2019/21	15714
Nigeria	2023/24	39050
Senegal	2023	16583
Sierra Leone	2019	15574
The Gambia	2019/20	11865

DHS is a nationally representative, multistage, stratified cluster sampling design conducted in more than 90 countries worldwide. This stratification and clustering design ensures that the DHS survey findings accurately reflect the target population of each country. DHS collects data on HIV/AIDS, nutrition, child health, child nutrition, reproductive health, family planning, marriage, fertility, and mortality. The sampling frame for each country is derived from a recent census and then stratified by region and residence (urban and rural), creating sampling strata. Initially, the enumeration areas (EAs) were selected in each stratum using probability proportional to size. Then, a listing of all households is conducted in each EA, where households are sampled with systematic random sampling. All eligible Women, men, and children in selected households will participate in the survey. Since the DHS survey has a complex sampling design, sample weight is given to consider for differential selection and nonresponse. For this study, we used DHS individual record (IR) files from the respective countries in SSA, yielding 467,965 women of reproductive age (15–49 years). Survey weights, clustering, and stratification variables were incorporated into the analysis to ensure representativeness and valid standard errors.

## Measurement of variables

### Target variable

This study focused on perceived healthcare accessibility as the target variable. In the DHS, women answered whether various factors posed significant barriers to accessing healthcare. We developed a composite outcome measure based on standard DHS questions, including: 1. Getting money for treatment (big problem/not a big problem), 2. Distance to a healthcare facility (big problem/not a big problem), 3. Transportation needs (big problem/not a big problem), and 4. Not wanting to go alone (big problem/not a big problem). If the woman faces at least one problem accessing healthcare, it is considered a big problem, coded as 1, and 0 otherwise.^34^

### Explainable variables

After reviewing various research,^5^^,^^16^^,^^35^, ^36^, ^37^, ^38^ features were extracted from the DHS dataset. These include Geographic Region, Maternal Age, Maternal Education Status, Residence, Wealth Index, Sex of Household Head, Maternal Working Status, Ownership of a Mobile Phone, Internet Use, Recent Birth History, Recent Healthcare Facility Visits, Health Insurance Status, Media Exposure, History of Abortion, and Contraceptive Use (Supplementary Table S1).

### Data preprocessing and management

**Figure 1:**
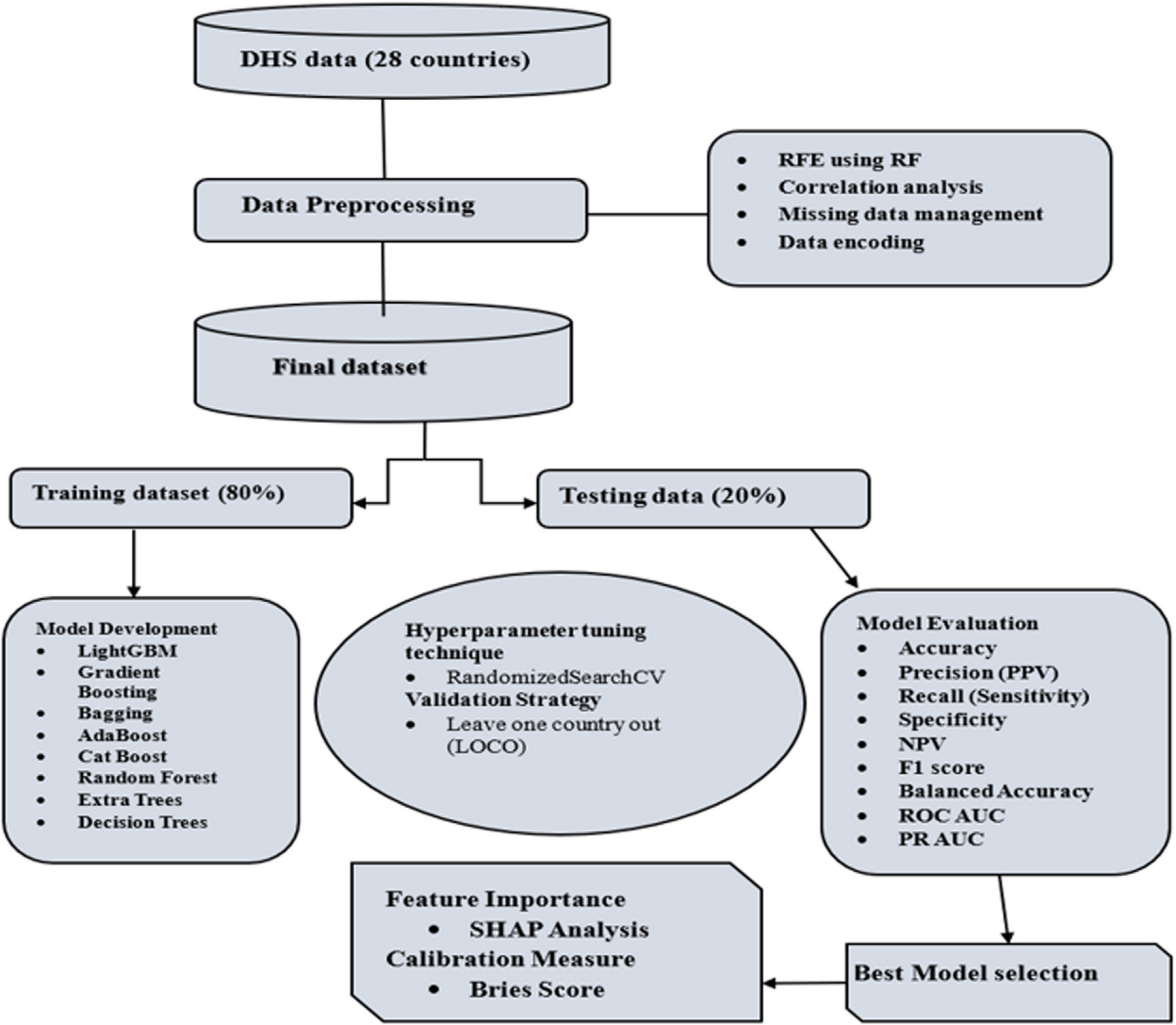
The workflow of methods.

As shown in Figure 1, the workflow starts by extracting IR files for DHS data from 28 SSA countries from the DHS website. Then Preprocessed by missing data management, data encoding, correlation analysis, and feature selection. The final dataset was split with 80/20 for the training and testing sets, respectively. Eight ML Models (LightGBM, Gradient Boosting, Bagging, AdaBoost, CatBoost, Random Forest, Extra Trees, and Decision Trees) were trained using RandomizedSearchCV for hyperparameter optimization and leave-one-country-out (LOCO) validation to assess generalizability. Accuracy, precision, recall (sensitivity), specificity, negative predictive value (NPV), F1 score, Balanced accuracy, ROC AUC, and PR AUC were used as evaluation metrics on the test dataset. Finally, the best model was selected, SHAP analysis was performed to identify top predictors, and calibration was assessed using the Brier score. The datasets were merged using Stat version 17 and exported to Python 3.14 for preprocessing, model development, and testing (Figure 1).

Kebede et al. A machine learning analysis of perceived healthcare access barriers among women of reproductive age in Sub-Saharan Africa. Am J Obstet Gynecol 2026.

### Correlation matrix and feature selection

**Figure 2:**
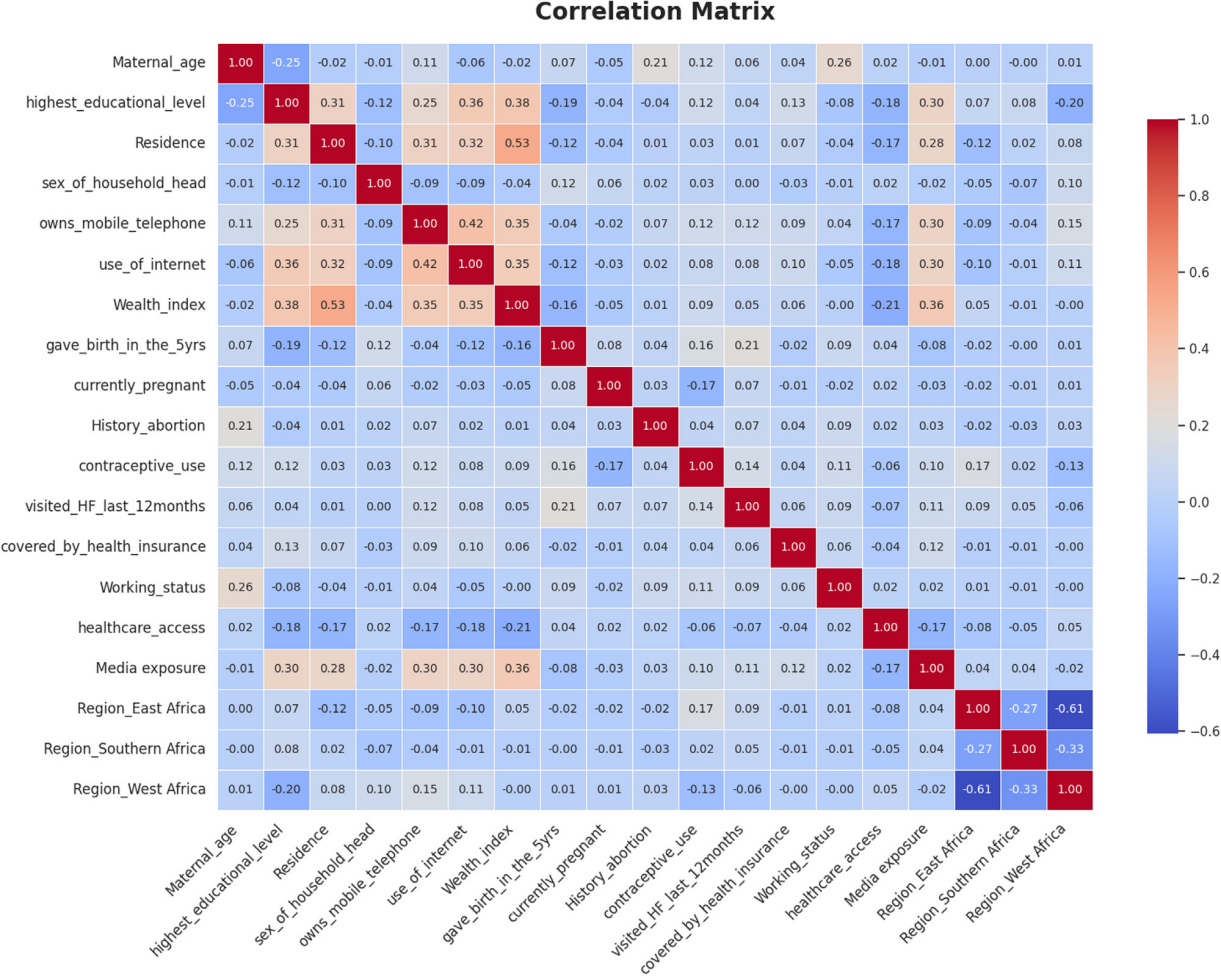
Correlation matrix heatmap.

To visualize the relationships among explainable features, we created a correlation matrix heatmap (Figure 2) that highlights both strong and weak correlations, making it easier to spot potentially redundant or complementary variables.

Kebede et al. A machine learning analysis of perceived healthcare access barriers among women of reproductive age in Sub-Saharan Africa. Am J Obstet Gynecol 2026.

**Figure 3:**
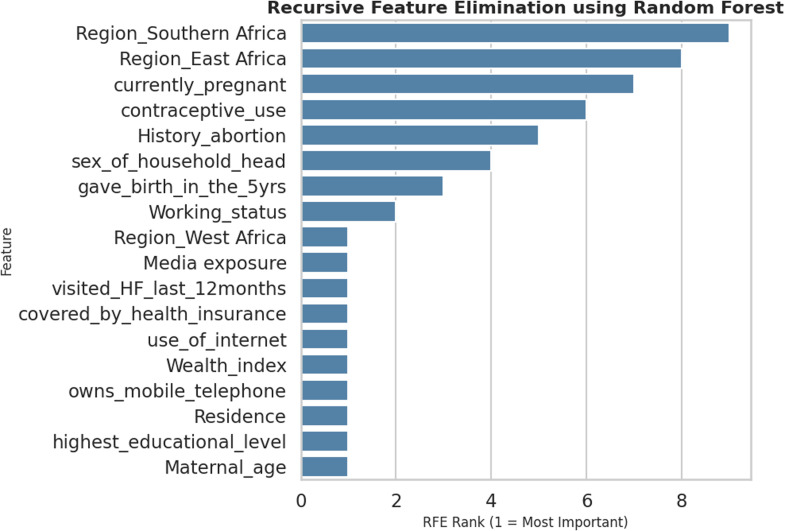
Feature selection using random forest embedded recursive feature elimination.

After a train-test split of the dataset, we then applied recursive feature elimination (RFE) with random forest classifiers exclusively on the training dataset to remove irrelevant features during training and testing model development, enhancing efficiency, preventing data leakage, ensuring unbiased evaluation of model performance, and interpretability (Figure 3). RFE is a method that iteratively assesses and discards less important features based on importance scores derived from the model, until only the most relevant variables remain.^39^ This process improves model performance, helps reduce overfitting by eliminating noise, and makes the model easier to interpret. With RFE, we selected the top 10 features used to train and test the model, which are: Women’s age, Women’s highest Education Level, Residence, owning a mobile telephone, internet use, Household Wealth index, recent visit to a health facility, health insurance status, working status, media exposure, and geographical region (West Africa) (Figure 3).

Kebede et al. A machine learning analysis of perceived healthcare access barriers among women of reproductive age in Sub-Saharan Africa. Am J Obstet Gynecol 2026.

### Handling imbalanced data

Class imbalance is a common challenge in ML that substantially affects model performance and introduces bias in predictions. A class-weighting approach was used to address class imbalance during model training. In the class weighting approach, higher weights were assigned to the minority class and lower weights to the majority class, increasing the penalty for biased classification of underrepresented instances and encouraging the model to pay greater attention to the minority class while preserving the original data distribution. This approach enables ML models to learn from available data more effectively because, unlike oversampling techniques, it doesn’t generate synthetic samples; it improves the model's ability to recognize minority patterns and increase generalizability.^40^^,^^41^

### Machine learning models

Eight machine learning models, including Boosting methods (Adaptive Boosting (Ada), Gradient Boosting Machine (GBM), Light Gradient Boosting Machine (LGBM)); Bagging techniques (Random Forest Classifier, Bagging classifier (Bagging), Extremely Randomized Trees, Categorical Boosting; and a Base Model (Decision Tree Classifier (DT)), were trained to predict perceived healthcare access barriers in SSA.

### Model training, validation, and evaluation

Model performance was measured using a leave-one-country-out (LOCO) validation strategy to assess the generalizability of MLs across different SSA countries. Data from the country was held out as an independent test set, while the remaining countries' data were used to train ML models, until every country had served as a testing once, thereby providing a robust evaluation model’s capacity to predict perceived healthcare access barriers in unseen countries.

Hyperparameter tuning was conducted using RandomizedSearchCV with 5-fold cross-validation on the training dataset within the LOCO iteration to enhance performance and prevent overfitting (Supplementary Table S2). The final evaluation on the test data employed standard metrics: Accuracy, Precision (PPV), Recall (Sensitivity), Specificity, Negative predictive value (NPV), F1-score, balanced Accuracy, Area under the ROC curve (ROC-AUC), and precision-recall AUC (PR-AUC), offering a robust assessment of model effectiveness by capturing both overall accuracy and the balance between sensitivity and specificity. Performance metrics were averaged across all LOCO iterations to obtain a robust and generalizable estimate of model performance. In addition, model calibration was further assessed using the Brier score and reliability curves to assess the agreement between predicted probabilities and observed outcomes. To quantify the uncertainty of model performance estimates, 95% Confidence intervals were computed using bootstrap resampling (Supplementary Table S3). Given the class imbalance, the model with the highest mean F1 score and ROC-AUC across countries, demonstrating good calibration and a low Brier score, was identified as the top performer for predicting perceived healthcare access barriers among women of reproductive age in SSA.

### Model interpretation

SHapley Additive exPlanations was used to identify and quantify the impact of key predictors on perceived healthcare access barriers among women of reproductive age. The SHAP values offered both overall and individual insights into feature importance, improving transparency and clarity regarding the factors influencing model predictions (1).^42^

## Results

Of the weighted 467,965 women of reproductive age in SSA included in the study, 53,303 (11.39%) respondents were from Central Africa, 152,847 (32.66%) from Eastern Africa, 60,258 (12.88%) from Southern Africa, and 201,557 (43.07%%) from Western Africa (Table 2).

**Table 2:** Socio-demographic, socioeconomic, and maternal characteristics of women of reproductive age in Sub-Saharan Africa.

**Variable**	**Category**	Weighted frequency	Weighted percentage (%)
Region	Central Africa	53,303	11.39%
	East Africa	152,847	32.66%
	Southern Africa	60,258	12.88%
	West Africa	201,557	43.07%
Maternal age	15–24	191,796	40.99%
	25–34	141,193	30.17%
	35–49	134,977	28.84%
Highest educational level	No Education	130,749	27.94%
	Primary Education	140,414	30.01%
	Secondary Education and above	196,803	42.05%
Residence	Urban	191,341	40.89%
	Rural	276,624	59.11%
Sex of household head	Male	334,573	71.50%
	Female	133,392	28.50%
Owns a mobile telephone	No	199,667	42.67%
	Yes	268,298	57.33%
Use of the internet	No	350,844	74.97%
	Yes	117,121	25.03%
Wealth index	Poor	167,281	35.75%
	Middle	90,525	19.34%
	Rich	210,159	44.91%
Currently pregnant	No	432,186	92.35%
	Yes	35,779	7.65%
History abortion	No	404,797	86.50%
	Yes	63,168	13.50%
Contraceptive use and intention	No	344,970	73.72%
	Yes	122,995	26.28%
Visited HF in the 4 last 12 months	No	212,307	45.37%
	Yes	255,658	54.63%
Covered by health insurance	No	418,825	89.50%
	Yes	49,140	10.50%
Working status	No	201,929	43.15%
	Yes	266,036	56.85%
Media exposure	No	227,168	48.54%
	Yes	240,797	51.46%
Gave birth in the past 5 years	No	234,124	50.03%
	Yes	233,841	49.97%

### Sociodemographic characteristics of participants

Most participants are aged 15 to 24, totaling 192,868 (40.99%). The majority of women (188,633; 40.3%) have completed secondary or higher education. Nearly two-thirds of women (286,767; 61.3%) come from rural areas. Among women, 333,103 (71.2%) live in male-headed households, and 262,330 (56.1%) own a mobile phone. However, only 106,542 women (22.8%) used the internet at least once in the past year. Most women, 405,689 (86.7%), have no history of abortion, and 349,074 (74.6%) do not use contraceptives or plan to. Additionally, 418,901 women (89.5%) lack health insurance, and half have no media exposure (Table 2).

### Prevalence of perceived healthcare access barriers among women in Sub-Saharan countries

The weighted prevalence of perceived barriers to healthcare access in SSA countries ranges from 17.8% (95% CI 17.0%-18.6%) in South Africa to with 72.1% (95% CI 71.6–72.7) in Malawi (Table 3).

**Table 3:** Prevalence of perceived barriers to healthcare access among women of reproductive age in Sub-Saharan African countries.

Country	Total sample size (weighted)	Unweighted perceived healthcare access barriers (95% CI)	Weighted perceived healthcare access barriers (95% CI)
South Africa	8514	18.1% (17.3%–18.9%)	17.8% (17.0%–18.6%)
Kenya	32156	29.2% (28.7%–29.7%)	27.2% (26.8%–27.7%)
Lesotho	6413	41.8% (40.6%–43.0%)	37.3% (36.1%–38.5%)
Zambia	13683	40.1% (39.3%–41.0%)	38.1% (37.3%–38.9%)
The Gambia	11865	45.5% (44.6%–46.4%)	42.7% (41.8%–43.6%)
Mali	20354	48.0% (47.3%–48.7%)	44.3% (43.6%–45.0%)
Liberia	8065	50.9% (49.8%–52.0%)	44.7% (43.6%–45.7%)
Rwanda	14634	48.8% (48.0%–49.6%)	48.6% (47.8%–49.4%)
Tanzania	15254	47.0% (46.3%–47.8%)	49.8% (49.0%–50.6%)
Mozambique	13183	46.7% (45.8%–47.5%)	49.8% (48.9%–50.6%)
Ghana	15014	56.9% (56.1%–57.7%)	53.6% (52.8%–54.4%)
Nigeria	39050	57.1% (56.6%–57.6%)	54.5% (54.0%–55.0%)
Gabon	11043	59.4% (58.5%–60.3%)	54.8% (53.9%–55.7%)
Uganda	18506	60.7% (60.0%–61.4%)	58.6% (57.9%–59.3%)
Benin	15928	60.2% (59.4%–60.9%)	60.4% (59.6%–61.1%)
Mauritania	15714	66.6% (65.8%–67.3%)	66.9% (66.1%–67.6%)
Cameroon	14677	67.4% (66.6%–68.1%)	67.2% (66.4%–67.9%)
Burkina Faso	17659	67.3% (66.6%–68.0%)	68.1% (67.4%–68.8%)
Guinea	10874	69.3% (68.4%–70.1%)	68.1% (67.2%–69.0%)
DR. Congo	27583	74.7% (74.2%–75.2%)	69.1% (68.5%–69.6%)
Madagascar	18869	70.7% (70.1%–71.4%)	70.0% (69.3%–70.6%)
Ethiopia	15683	64.3% (63.5%–65.0%)	70.0% (69.3%–70.7%)
Senegal	16583	70.2% (69.5%–70.9%)	70.1% (69.4%–70.8%)
Angola	14379	69.2% (68.4%–69.9%)	70.2% (69.5%–71.0%)
Burundi	17269	69.8% (69.1%–70.5%)	70.5% (69.9%–71.2%)
Côte d’Ivoire	14877	73.6% (72.9%–74.3%)	71.4% (70.6%–72.1%)
Sierra Leone	15574	74.2% (73.5%–74.9%)	71.9% (71.2%–72.6%)
Malawi	24562	66.9% (66.3%–67.5%)	72.1% (71.6%–72.7%)

### Model performance metrics

Among the evaluated models, evaluation metrics showed generally comparable results across algorithms, with minimal variation in accuracy and discriminative capacity. The accuracy of the evaluated models ranged from 64.1% to 65.2%, indicating consistent predictive performance. LightGBM achieved the best overall predictive performance with accuracy: 65.2%, sensitivity: 79.7%, precision: 67.1%, and F1 score: 72.8%. Moreover, lightGBM demonstrated the highest ROC AUC (68.2%) and PR AUC (72.9%) among all evaluated models, making it the most balanced model for effectively detecting positive cases. Gradient Boosting followed with comparable recall (80.6%) and F1-score (72.9%) results, but its specificity (42.7%), balanced accuracy (61.7%), ROC AUC (67.7%), and PR AUC (72.3%) were slightly lower. Ensemble methods such as Random Forest, Extra Trees, and CatBoost also performed competitively, showing slightly higher precision but lower recall, which indicates they are more conservative in predicting positives. Overall, LightGBM provided the best overall balance across multiple evaluation metrics, with the highest precision, ROC-AUC, and PR-AUC (Table 4 and Supplementary Table S3).

**Table 4:** Model comparison evaluation metrics.

Model	Accuracy	Recall (sensitivity)	Specificity	Precision (PPV)	NPV	F1-score	Balanced accuracy	ROC-AUC	PR-AUC
**LightGBM**	65.2%	79.7%	44.6%	67.1%	60.8%	72.8%	62.2%	68.2%	72.9%
**GradientBoosting**	64.9%	80.6%	42.7%	66.6%	60.9%	72.9%	61.7%	67.7%	72.3%
**Bagging**	64.9%	78.3%	46.0%	67.2%	60.0%	72.4%	62.1%	67.7%	72.3%
**AdaBoost**	64.1%	78.9%	43.1%	66.2%	59.1%	72.0%	61.0%	66.5%	71.0%
**CatBoost**	65.1%	78.9%	45.6%	67.3%	60.4%	72.6%	62.3%	68.1%	72.8%
**RandomForest**	65.0%	78.3%	46.1%	67.3%	60.0%	72.4%	62.2%	67.8%	72.4%
**ExtraTrees**	65.0%	78.1%	46.4%	67.3%	59.9%	72.3%	62.2%	67.8%	72.4%
**DecisionTree**	65.0%	78.1%	46.4%	67.3%	59.9%	72.3%	62.2%	67.8%	72.3%

### Calibration assessment

**Figure 4:**
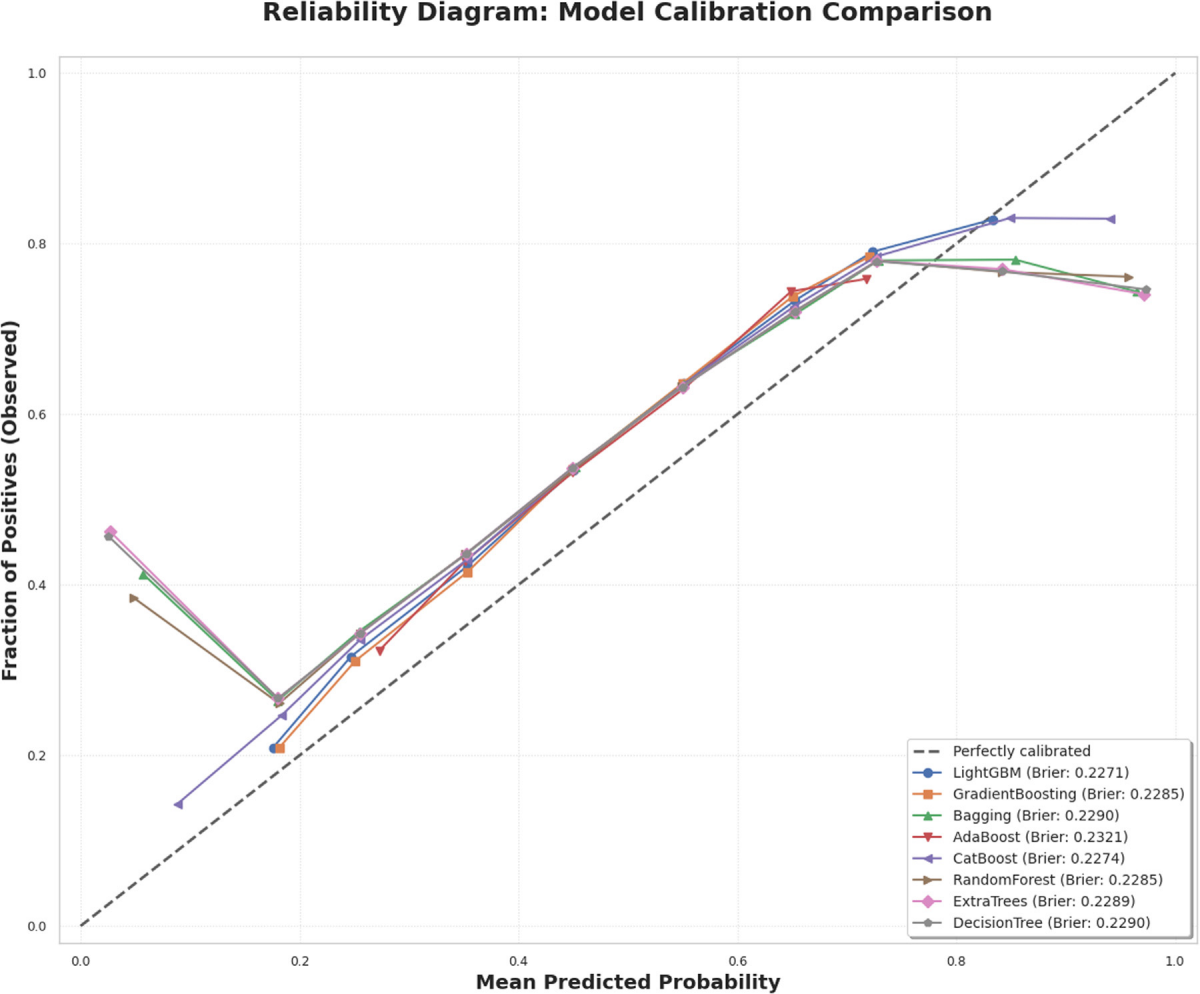
Reliability diagram and Brier score.

According to the calibration measure for perceived healthcare access barriers in Figure 4, all eight ML models showed comparably good agreement between predicted probabilities and observed outcomes. LightGBM exhibited the best calibration measure with the lowest Brier score (0.2271), which indicates the most accurate probability estimate across all models. Catboost (0.2274), Gradient Boosting, and Random Forest (0.2289), Bagging, and Decision Tree (0.2290) also resulted in comparable performance. But Adaboost, with a Brier score of 0.2321, had slightly poor calibration (Figure 4).

Kebede et al. A machine learning analysis of perceived healthcare access barriers among women of reproductive age in Sub-Saharan Africa. Am J Obstet Gynecol 2026.

### SHAP analysis

**Figure 5:**
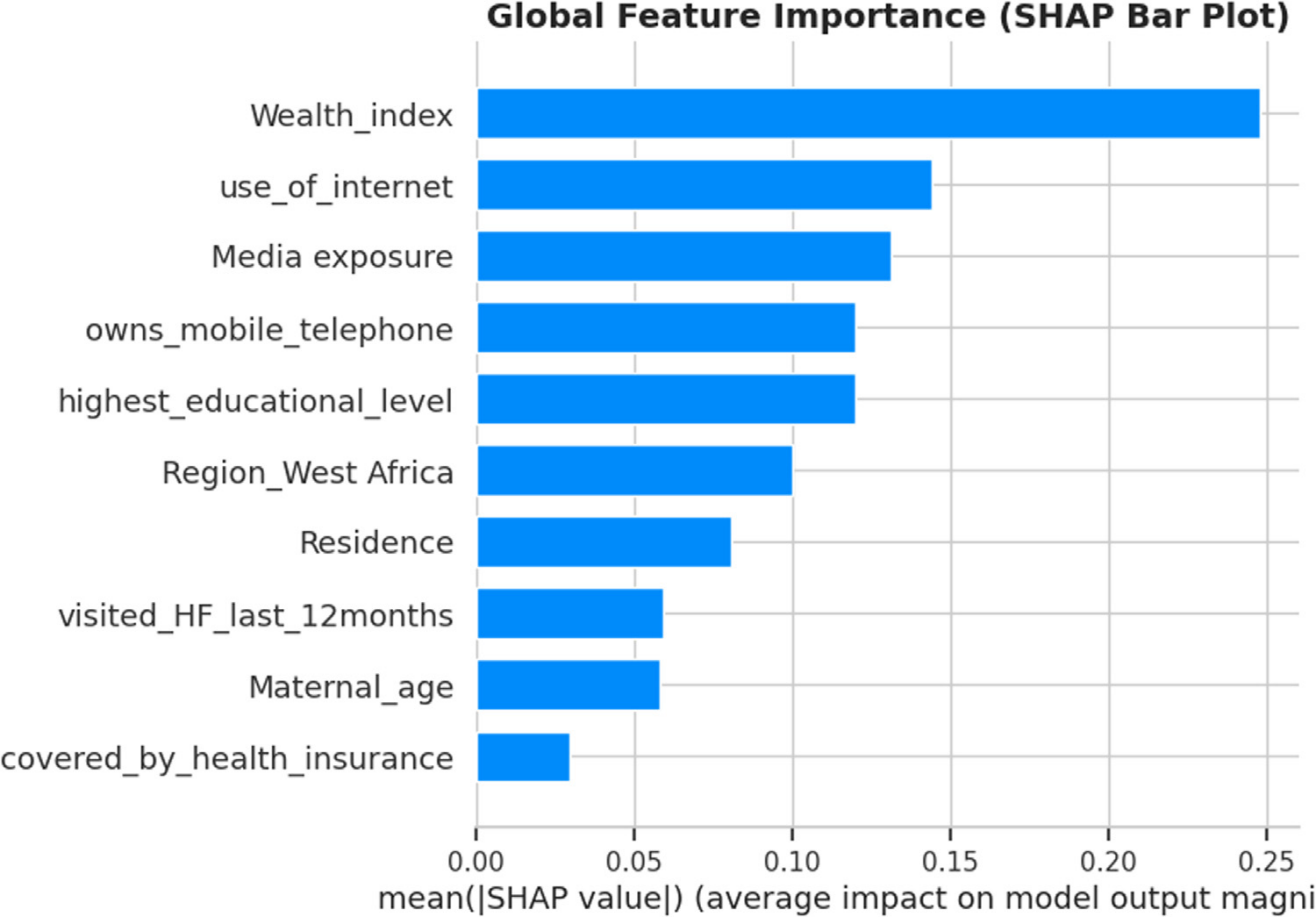
SHAP bar plot.

**Figure 6:**
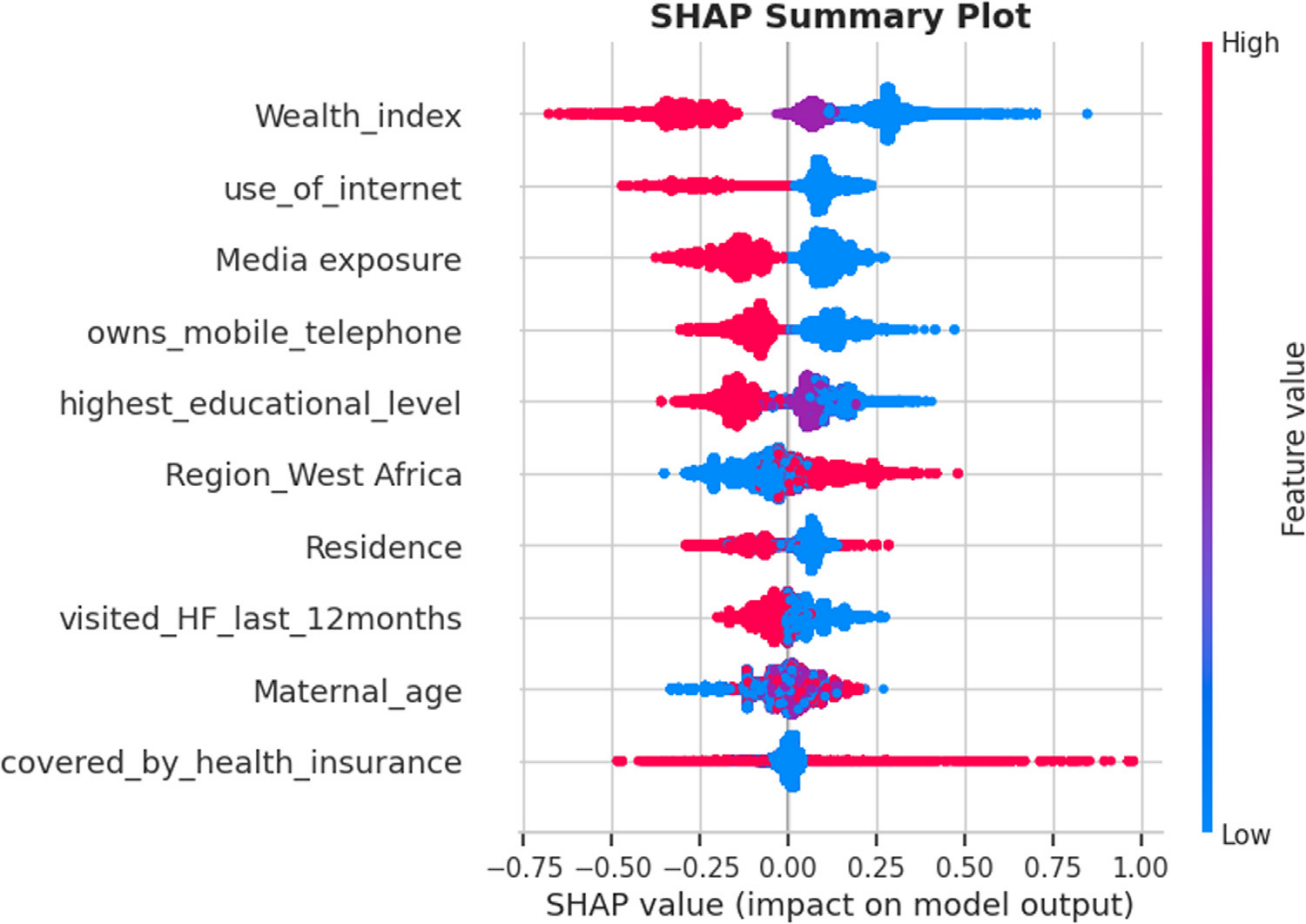
SHAP beeswarm plot.

The SHAP analysis shows that the socioeconomic and information access variables are the key determinants influencing the prediction of perceived healthcare access barriers among women of reproductive age in SSA. As shown in Figure 5, the Wealth Index, internet use, media exposure, ownership of mobile telephone, women’s education level, geographic region (West Africa), residence, history of health facility visits (within 12 months), women’s age, and health insurance coverage were the most significant factors. Women with lower wealth were more likely to face problems with healthcare access, while healthier women were less likely to do so. Similarly, higher media exposure, internet use, Mobile Phone ownership, and higher educational level were associated with reduced perceived healthcare access barriers, indicating the role of information and communication in improving access. Regional variation was also important, with residence in West Africa increasing the perceived healthcare access barriers. Other factors, such as recent health facility visit, maternal age, residence, and health insurance coverage, have smaller but notable influences (Figure 6).

Kebede et al. A machine learning analysis of perceived healthcare access barriers among women of reproductive age in Sub-Saharan Africa. Am J Obstet Gynecol 2026.

Kebede et al. A machine learning analysis of perceived healthcare access barriers among women of reproductive age in Sub-Saharan Africa. Am J Obstet Gynecol 2026.

## Discussion

The current study used a large, pooled, and nationally representative dataset from 28 Sub-Saharan African countries to examine the determinants of perceived healthcare access barriersamong women of reproductive age using a robust ML framework. Our analysis returned three major findings: first, the overall pooled prevalence of perceived healthcare access barriers in SSA is ranges from 17.8% (95% CI, 17.0%–18.6%) in South Africa to 72.1% (95% CI, 71.6%–72.7%), though with marked regional and country variations; second, among the eight ML models compared, the LightGBM classifier yielded the most balanced and efficient predictive performance with Accuracy: 65.2, F1-score: 72.8%, ROC-AUC: 68.2%, and PR-AUC: 72.9%; finally, the interpretability analysis using SHAP identified socioeconomic status (Wealth Index), information and communication factors (Media Exposure, Internet Use, Mobile Phone Ownership), and educational attainment as the most powerful predictors of perceived healthcare access.

The high overall prevalence of perceived healthcare access problems indicates a persistent and critical barrier to attaining Universal Health Coverage (UHC) and the health-related SDGs in SSA. That more than half of the women reported at least one major barrier to care is consistent with previous multicountry studies that have documented the pervasive nature of these challenges.^5^^,^^43^ The marked regional variation probably reflects deeper structural inequities, including weaker health systems, greater political instability, and higher levels of poverty in these regions compared to Southern and Eastern Africa. The case of the Malawi, which reported the highest prevalence (72.1%), exemplifies a setting where conflict and fragile infrastructure compound existing access barriers.

The excellence of the LightGBM model, an advanced tree-based boosting algorithm, signifies the importance of ML in managing very complex and high-dimensional demographic and health survey data. Capable of modelling nonlinear relationships and complex interactions, it outperformed the other ensemble methods and the base Decision Tree classifier. High recall among top-performing models is what is most valuable from the perspective of public health because it indicates a strong capacity to correctly identify the majority of women genuinely having access problems. This minimizes false negatives to make sure interventions can be effectively provided to those in most need. Facilitated by SHAP analysis, the interpretability of our model goes beyond simple prediction by providing actionable insights into the root causes of access barriers. The dominance of the Wealth Index as the most important predictor serves as a sobering reminder that poverty remains the single greatest obstacle to healthcare in SSA. This finding is consistent with the large volume of literature linking economic disadvantage to catastrophic health expenditures and forgone care.^44^, ^45^, ^46^ A strong protective effect of Media Exposure, Internet Use, and Mobile Phone Ownership underlines an important and evolving dimension of healthcare access. These factors likely act through the improvement of health literacy, enabling women to seek out information on services, facilitating communication with providers and peer networks, hence reducing information asymmetry.^47^ Similarly, educational attainment reduces access problems by reinforcing the well-established link between improved health-seeking behavior, empowerment, and the ability of women to navigate complex health systems.^48^, ^49^, ^50^, ^51^ Other correlates, including recent health facility visits, health insurance, and place of residence (rural/urban), were also significant predictors in our model, although at a relatively lower magnitude. The association between health facility visits and access problems is likely to be bidirectional, with difficulties in accessing services preventing visits, but, on the other hand, a recent visit could also raise awareness among women about existing barriers. The relatively modest effect of health insurance, despite its theoretical importance, may reflect the generally very low coverage, only 10.5% in our sample, and the limited depth of coverage that the existing schemes may offer in most SSA countries.^52^

These insights from the analysis, therefore, indicate a multifaceted, evidence-based approach that policymakers can take in efforts towards dismantling health access barriers for women in Sub-Saharan Africa. In sum, there should be increased multisectoral efforts for poverty alleviation and economic empowerment through targeted cash transfer programs, microfinance initiatives, and livelihood support among the poorest households. Besides these, policies should facilitate digital inclusion through the advancement of digital literacy, ensuring affordable access to mobile phones and the internet, especially in rural areas; these technologies can be leveraged for mHealth interventions such as appointment reminders, health information dissemination, and teleconsultations to directly mitigate access barriers. Further, sustained investment in girls' education, especially at the secondary and higher levels, is important in building long-term health agency and access. Finally, beyond traditional approaches, the predictive model developed in this study could be operationalized within national health management information systems to allow district health managers to proactively identify clusters of high-risk women, thus enabling timely and targeted outreach and support.

### Limitations of the study

There are several limitations to this study. First, the cross-sectional nature of the DHS data precludes causal inference from our findings; we can only identify associations. Second, the outcome variable is self-reported perceptions of problems, which may be vulnerable to reporting bias and may not necessarily align with more objective measures of access. Third, although we have an extensive list of covariates, not all confounders can be captured by the data, especially unmeasured factors such as quality of care, more specific cultural norms, or detailed geographic barriers, such as terrain. Finally, although the SMOTE addressed class imbalance, the robustness of the predictive accuracy of the models suggests that a significant proportion of the variance in healthcare access is not explained by the variables used, indicating both the complexity of the phenomenon and the influence of latent variables.

## Conclusion

This study, therefore, reveals that ML, and more importantly, interpretable models such as LightGBM coupled with SHAP, offer a powerful, fine-grained approach to the multilayered problem of perceived barriers toin SSA. We confirm that socio-economic and communication-related factors are, overall, the leading determinants of access barriers for women. The high prevalence and identifiable risk profiles undergird the urgent requirement for context-specific, multipronged strategies to address the root causes of inequity. Future research should integrate longitudinal data to establish causality and incorporate qualitative methods to deepen the interpretation of the predictive features identified here, thereby forging a path toward more equitable and effective health systems in Sub-Saharan Africa.

## Ethical approval and consent to participate

ICF International and the respective country-level Institutional Review Boards obtained ethical approval for the primary DHS data collection. Permission to access and use the datasets for this study was granted by the DHS Program (www.dhsprogram.com). As this research involved the secondary analysis of existing, de-identified, publicly available data, no additional Institutional Review Board (IRB) approval was sought. All data were treated with strict confidentiality. Informed consent, confidentiality, anonymity, and participant privacy were rigorously maintained by the DHS program during the original data collection. The research is conducted in accordance with the Declaration of Helsinki.

## Availability of data and materials

All data are available at www.dhsprogram.com.

## CRediT authorship contribution statement

**Fentahun Bikale Kebede:** Writing – review & editing, Writing – original draft, Visualization, Validation, Supervision, Software, Resources, Project administration, Methodology, Investigation, Funding acquisition, Formal analysis, Data curation, Conceptualization. **Abraham Keffale Mengistu:** Writing – review & editing, Writing – original draft, Visualization, Data curation.

## Appendix Supplementary materials

Image, application 1

## Footnotes

Supplementary material associated with this article can be found in the online version at doi:10.1016/j.xagr.2026.100668.
