# Supplementary material for: A machine learning analysis of perceived healthcare access barriers among women of reproductive age in Sub-Saharan Africa: using demographic and health surveys
Source: AJOG Glob Rep. 2026 Jul 3;6(3):100668. doi: 10.1016/j.xagr.2026.100668 (PMC13453436; doi:10.1016/j.xagr.2026.100668)
Supplement: Supplementary file 1 [file mmc1.docx]

**Supplementary files**

**Supplementary Table S1:** Variables, their categories, definition, and missingness in percent

| **Variable** | **DHS Variables** | **Recoding/Definition** | **Category** | **Missingness**  **Percentage** |
| --- | --- | --- | --- | --- |
| Region | V000 | The geographical region of the country included in the study, according to the World Bank | Central Africa, East Africa, Southern Africa, West Africa | 0.00% |
| Maternal Age | V012 | Age of the respondent at the time of the survey. | 15–24 years, 25–34 years, 35–49 years | 1.25% |
| Highest Educational Level | V106 | Highest educational level attained | No Education, Primary Education, Secondary Education, and Above | 0.00% |
| Residence | V025 | Type of Place of residence | Urban, Rural | 0.00% |
| Sex of Household Head | V151 | Sex of the household head | Male, Female | 0.00% |
| Owns a Mobile Telephone | V169A | Ownership of a mobile telephone | No, Yes | 0.00% |
| Use of the Internet | V171A | Ever used the internet | No, Yes | 0.00% |
| Wealth Index | V190 | Composite measure of a household's cumulative living standard | Poor, Middle, Rich | 0.00% |
| Currently Pregnant | V213 | Pregnancy status at the time of the survey | No, Yes | 1.25% |
| History of Abortion | V228 | History of a terminated pregnancy | No, Yes | 1.25% |
| Contraceptive Use | V313 | Status of contraceptive use | No, Yes | 1.25% |
| Visited Health Facility in the Last 12 Months | V394 | Visited a health facility during the last 12 months | No, Yes | 5.80% |
| Covered by Health Insurance | V481 | Health insurance coverage status | No, Yes | 9.40% |
| Working Status | V714 | Employment status at the time of the survey | No, Yes | 1.00% |
| Media Exposure | Derived from v157, v158, and v159 | Exposure to newspapers, radio, or television at least once a week | No, Yes | 0.00% |
| Gave Birth in the Last 5 Years | V208 |  | No, Yes | 1.25% |

**Supplementary Table S2:** Hyperparameter grids used for RandomizedSearchCV, and fine-tune parameters

| **Model** | **Hyperparameter Grid** | **Fine-tuned parameters** |
| --- | --- | --- |
| LightGBM | n_estimators = {100, 200}; learning_rate = {0.01, 0.1}; num_leaves = {31, 50} | n_estimators = 200; learning_rate = 0.1; num_leaves = 50 |
| Gradient Boosting | n_estimators = {100, 200}; learning_rate = {0.01, 0.1}; max_depth = {3, 5} | n_estimators = 200; learning_rate = 0.1; max_depth = 3 |
| Bagging | n_estimators = {10, 50} | n_estimators = 50 |
| AdaBoost | n_estimators = {50, 100}; learning_rate = {0.1, 1.0} | n_estimators = 100; learning_rate = 0.1 |
| CatBoost | iterations = {100, 200}; depth = {4, 6}; learning_rate = {0.01, 0.1} | iterations = 200; depth = 6; learning_rate = 0.1 |
| Random Forest | n_estimators = {100, 200}; max_depth = {10, 20} | n_estimators = 200; max_depth = 20 |
| Extra Trees | n_estimators = {100, 200}; max_depth = {10, 20} | n_estimators = 200; max_depth = 10 |
| Decision Tree | max_depth = {5, 10, None} | max_depth = 10 |

**Supplementary Table S3:** Evaluation Metrics with their 95% Confidence Interval

| **Model** | **Accuracy (95% CI)** | **Sensitivity (95% CI)** | **Specificity (95% CI)** | **PPV (95% CI)** | **NPV (95% CI)** | **F1-score (95% CI)** | **Balanced Accuracy (95% CI)** | **ROC-AUC (95% CI)** | **PR-AUC (95% CI)** |
| --- | --- | --- | --- | --- | --- | --- | --- | --- | --- |
| LightGBM | 0.652 (0.649-0.654) | 0.797 (0.794-0.799) | 0.446 (0.441-0.452) | 0.671 (0.668-0.674) | 0.608 (0.603-0.613) | 0.728 (0.726-0.731) | 0.622 (0.619-0.625) | 0.682 (0.678-0.685) | 0.729 (0.724-0.733) |
| GradientBoosting | 0.649 (0.647-0.652) | 0.806 (0.803-0.809) | 0.427 (0.423-0.432) | 0.666 (0.663-0.669) | 0.609 (0.605-0.614) | 0.729 (0.727-0.732) | 0.617 (0.614-0.619) | 0.677 (0.673-0.680) | 0.723 (0.718-0.727) |
| Bagging | 0.649 (0.646-0.653) | 0.783 (0.781-0.786) | 0.460 (0.454-0.464) | 0.672 (0.669-0.676) | 0.600 (0.595-0.605) | 0.724 (0.721-0.727) | 0.621 (0.618-0.625) | 0.677 (0.674-0.681) | 0.723 (0.718-0.727) |
| AdaBoost | 0.641 (0.638-0.643) | 0.789 (0.786-0.792) | 0.431 (0.426-0.436) | 0.662 (0.659-0.666) | 0.591 (0.586-0.596) | 0.720 (0.718-0.723) | 0.610 (0.608-0.613) | 0.665 (0.662-0.669) | 0.710 (0.705-0.715) |
| CatBoost | 0.651 (0.649-0.654) | 0.789 (0.786-0.791) | 0.456 (0.451-0.462) | 0.673 (0.669-0.676) | 0.604 (0.600-0.609) | 0.726 (0.724-0.729) | 0.623 (0.620-0.626) | 0.681 (0.677-0.685) | 0.728 (0.724-0.732) |
| RandomForest | 0.650 (0.647-0.653) | 0.783 (0.780-0.786) | 0.461 (0.455-0.466) | 0.673 (0.670-0.676) | 0.600 (0.595-0.606) | 0.724 (0.721-0.727) | 0.622 (0.619-0.625) | 0.678 (0.675-0.682) | 0.724 (0.719-0.728) |
| ExtraTrees | 0.650 (0.647-0.654) | 0.781 (0.783-0.789) | 0.464 (0.451-0.462) | 0.673 (0.669-0.675) | 0.599 (0.596-0.607) | 0.723 (0.722-0.728) | 0.622 (0.619-0.625) | 0.678 (0.675-0.682) | 0.724 (0.719-0.728) |
| DecisionTree | 0.650 (0.647-0.654) | 0.781 (0.783-0.788) | 0.464 (0.451-0.462) | 0.673 (0.669-0.675) | 0.599 (0.596-0.607) | 0.723 (0.722-0.727) | 0.622 (0.619-0.625) | 0.678 (0.674-0.681) | 0.723 (0.719-0.727) |
